# Harnessing Trichoderma Mycoparasitism as a Tool in the Management of Soil Dwelling Plant Pathogens

**DOI:** 10.1007/s00248-024-02472-2

**Published:** 2024-12-21

**Authors:** Srishti Singh, Alok Kumar Singh, Bhubaneswar Pradhan, Sudipta Tripathi, Kewat Sanjay Kumar, Sasmita Chand, Prangya Ranjan Rout, Muhammad Kashif Shahid

**Affiliations:** 1 Department of Botany, CMP Degree College, University of Allahabad, Uttar Pradesh, Prayagraj, 211002 India; 2 Division of Agricultural Biotechnology, School of Agriculture and Rural Development, Ramakrishna Mission Vivekananda Educational and Research Institute, Narendrapur Campus, Kolkata, 700103 West Bengal India; 3 School of Environment and Disaster Management, Ramakrishna Mission Vivekananda Educational and Research Institute, Narendrapur Campus Kolkata, Kolkata, 700103 West Bengal India; 4 Department of Botany, University of Allahabad, Uttar Pradesh, Prayagraj, 211002 India; 5 Manipal School of Architecture and Planning, Manipal Academy of Higher Education, Manipal, Karnataka 576104 India; 6 Department of Biotechnology, Dr B Ambedkar National Institute of Technology Jalandhar, Punjab, 144011 India; 7 Faculty of Civil and Architecture, National Polytechnic Institute of Cambodia (NPIC), Phnom Penh, 12409 Cambodia

**Keywords:** *Trichoderma*, Soil fungi, Biological control, Plant pathogens, Sustainable crop production

## Abstract

Maintaining and enhancing agricultural productivity for food security while preserving the ecology and environment from the harmful effects of toxicants is the main challenge in modern monoculture farming systems. Microbial biological agents can be a promising substitute for traditional synthetic pesticides to manage plant diseases. Trichoderma spp. are soil-dwelling ascomycete fungi and are common biocontrol agents against diverse phytopathogens. Trichoderma-based biocontrol techniques can regulate and control soil-borne plant diseases through mechanisms such as mycoparasitism, the production of antibiotics and hydrolytic enzymes, rhizo-sphere competence, the effective competition for available resources, induction of plant resistance and facilitation of plant growth. Numerous secondary metabolites produced by Trichoderma spp. are reported to prevent the development of soil-borne plant disease. Thus, Trichoderma spp. may have direct and indirect biological impacts on the targeted plant pathogens. Furthermore, this review discusses the convenient implications and challenges of applying Trichoderma-based strategies in agricultural settings. Overall, the assessment underscores the potential of Trichoderma as a sustainable and effective tool for mitigating soil-borne pathogens, highlighting avenues for future research and applications.

## Introduction

Near 2050, there will be 9.1 billion people on the planet, meaning that overall food production must increase by about 70% for food security [1]. Plant diseases have been a source of worry for humankind ever since the development of agriculture. These diseases played a significant part in the depletion of natural resources and were responsible for 16% of global crop yield losses [2]. Soil-borne plant pathogens economically affect crop production in tropical, subtropical, and temperate regions [3]. There are currently 2 million tons of pesticides used worldwide, with herbicides making up 47.5% of usage, insecticides making up 29.5%, fungicides making up 17.5%, and other pesticides making up the remaining 5.5% [4]. The top 10 countries in the world that use pesticides are China, the USA, Argentina, Thailand, Brazil, Italy, France, Canada, Japan, and India. It was observed that pesticide consumption worldwide increased by 20% over the past ten years [5]. Agricultural chemical pesticides are crucial for emerging nations to protect crops from insect pest attacks and increase crop yields. Pesticides can boost agricultural yield via direct control of phytopathogens. Still, their residual toxicity harms the environment, non-target organisms, biodiversity, food chain, human health, and food safety. Recent research on the environmental fate of chemical pesticides on soil, land, water, and living beings has been spurred by worry about the environment and human health [6]. Therefore, an urgent need is to explore and exploit biological control agents to manage phytopathogenic infections as an alternate and effective strategy.

Biocontrol is the term used to describe the function of naturally existing organisms in integrated pest management in reducing the number of plant pests [7]. Trichoderma species are the basis for more than 60% of the biofungicides currently licensed worldwide, making them the most effective biofungicides used in contemporary agriculture [8]. These fungi live in soil mostly confined to rhizospheric regions of plants. They colonize plant material like grains, leaves, and roots [9]. Trichoderma is the market leader for fungal bio-control agents on a global scale [10]. Due to its dual ability to prevent disease and act as soil compost, it has gained a unique place in agriculture as a potent biocontrol agent, plant growth stimulant, and soil fertility improver. Due to its rhizosphere competence, competitive saprophytic ability, capacity to manufacture or induce hormone production in plants, ability to release nutrients from the soil, and ability to increase root system architectural development, it acts as an effective plant growth promoting fungi (PGPF) [11]. Trichoderma can produce a variety of fungal enzymes, such as chitinases, glucanases, cellulases, and hemicellulases, responsible for the toxic action of fungi against soil-borne plant pathogens [12]. These enzymes are also used in the postharvest disease management of papaya, apple, tomato, pear, mango, banana, potato, and berries [13]. This genus has nine species, initially described in 1969 by Rifai and Webster [14]. The genus was further divided into five divisions based on conidiophore branching by Bissett (1991). *Trichoderma harzianum* and *T. viride* are most frequently used as bio-control agents. The present review attempts to analyze and evaluate the biological control potential of Trichoderma spp. in managing fungal plant pathogens.

### Trichoderma: Classification, Characteristics, and Benefits as a Biocontrol Agent

Trichoderma represents the asexual stage (anamorph), whereas *Hypocrea* corresponds to the sexual stage (teleomorph), according to their taxonomic classification [15]. In its asexual form, it falls under the division Deuteromycotina, while in the sexual stage, it is classified under Ascomycotina.

Trichoderma species (spp.) are widely distributed across agricultural regions in all climatic zones, thriving at temperatures ranging from 25 and 30° C, due to their saprophytic nature [16, 17]. For instance, *Trichoderma viride* and *Trichoderma polysporum* prefer mild temperatures, while *Trichoderma harzianum* prefers warm climatic conditions. These fungi are easily identifiable due to their unique smells, attributed to the volatile compound δ-lactone 6-pentyl-α-pyrone (6-PP), [18]. With their competitive saprophytic ability (CSA), Trichoderma spp. are also found in the rhizosphere of plants, where they induce systemic resistance against diseases and enhance plant growth and development [19]. Trichoderma spp. utilize a range of substances, including carbon and nitrogen sources for their sporulation activity [20], and produce abundant powdery masses with green conidia [21]. While the presence of *Trichoderma citrinoviride* has been reported in Southeast Asia, it is yet to be discovered in India [22]. Most of the Trichoderma spp. prefer acidic environments, however they can adapt to a wide pH ranging from 2.0 to 13.0 [23]. Typically, the conidial color morphology of Trichoderma spp. is green but can be grey, white, and yellow, depending on the species [24]. The dominant saprophytic ability of these fungi allows them to compete with other soil organisms and colonize plant roots effectively. Additionally, these fungi also produce secondary metabolites and enzymes that promote plant growth as well as enhance disease resistance against pathogens [25].

### Identification of Trichoderma Isolates

The genus Trichoderma includes a diverse range of fungi that exhibit a range of colony morphologies depending on the culture media used for their growth. For instance, when grown on potato dextrose agar (PDA) at 28 °C for seven days, Trichoderma cultures from soil samples displayed green pigmented colonies. Conversely, colonies from rhizospheric isolates grown at 25 °C and 30 °C appeared pale or yellowish, exhibiting rapid growth and conidia dispersion. The fungi can also be identified based on the arrangement of conidia and the phialides, which are projections of the conidiophores. Phialides, of these fungi in particular, were observed to be ellipsoidal, oblong, and bowling pinshaped [26]. Additionally, a study by Sekhar et al. (2017) reported that ten isolates from the rhizosphere of groundnuts exhibited various morphological and microscopic traits, including colony color, reverse color, and the shape and features of conidia, phialides, and conidiophores [27].

### Culture Media for Trichoderma

#### Selective Media for Trichoderma (TSM)

Trichoderma selective medium (TSM) is recognized as the gold standard for the quantitative separation of Trichoderma spp. from the soil. To grow quickly and sporulate, the fungus contains low glucose-specific fungal inhibitors, including pentachloronitrobenzene, p-dimethyl amino benzene, diazo sodium sulfonate, and rose bengal. At the same time, chloramphenicol is used to stop bacterial development [28].

##### Trichoderma Selective Media (TSM)

The ingredients and amounts required for Trichoderma selective medium are MgSO_4_∙7H_2_O (0.2 g), K_2_HPO_4_ (0.9 g), KCl (0.15 g), NH_4_NO_3_ (1.0 g), glucose (3.0 g), rose bengal (0.15 g), agar (20 g), chloramphenicol (0.25 g), p-dimethyl amino benzene diazo sodium sulfonate (0.3 g), pentachloro nitrobenzene (0.2 g), distilled water (1.0 L). For media preparation, the ingredients are mixed properly and then autoclaved for 15 min at 121 °C. The mixture is then supplemented with 0.25 g of chloramphenicol and 0.2 g of pentachloro nitrobenzene. To avoid solidification, the media should be maintained or stored at 45 °C.

##### *Trichoderma harzianum* Selective Medium (THSM)

The ingredients and amounts required for *T. harzianum* selective medium are the same as those mentioned for TSM including the media preparation procedure. However, antimicrobial agents such as chloramphenicol, streptomycin, quintozene, and propamocarb are added to the medium to isolate a pure colony of Trichoderma sp. [29]. For instance, the media after autoclaving are supplemented with 0.25 g of chloramphenicol, 9.0 ml of streptomycin, 1.2 ml of propamocarb, and 0.2 g of quintozene. The use of THSM makes the comparison of aggressive and non-aggressive Trichoderma groups possible.

## Biological Control Strategies Employed by Trichoderma spp.

**Fig. 1:**
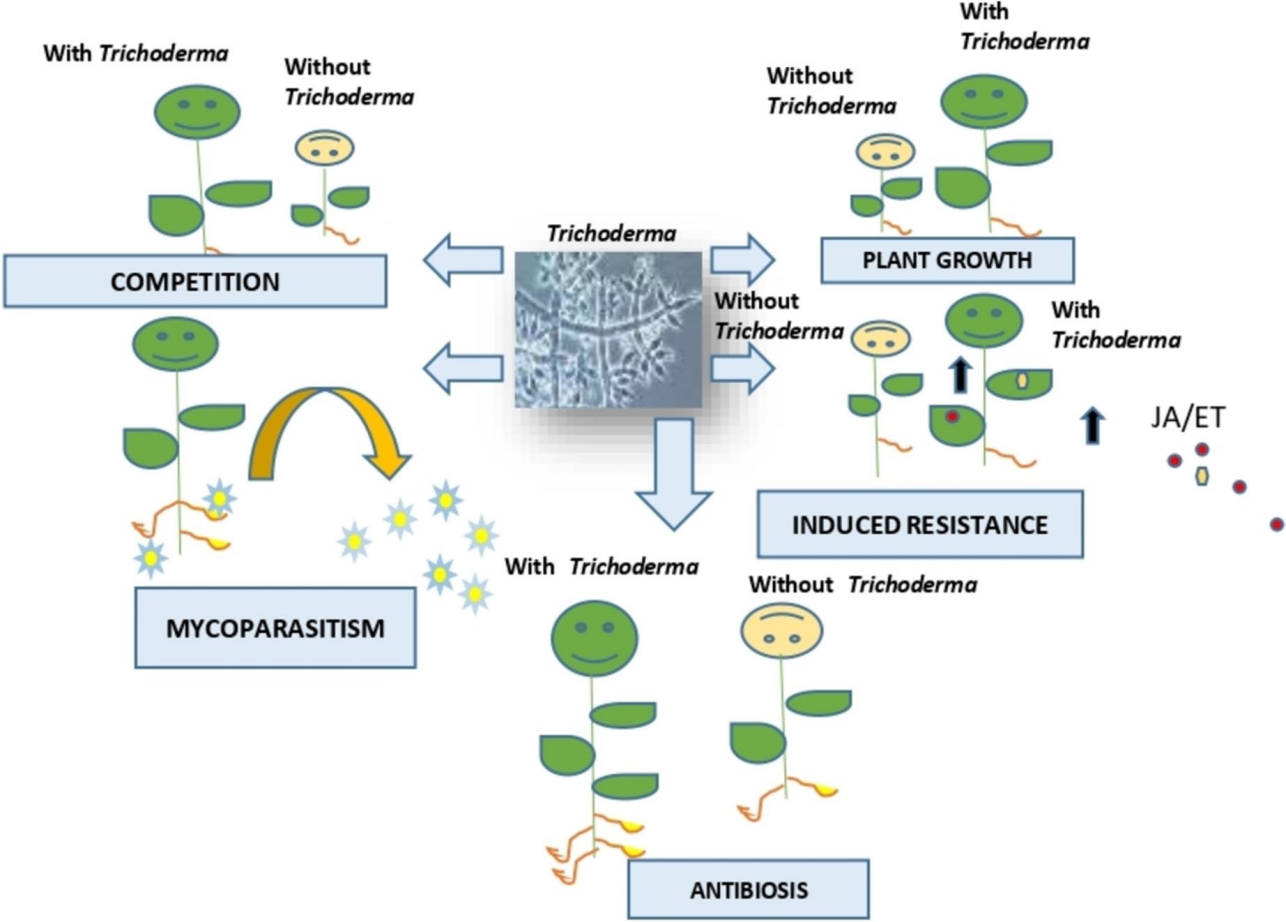
The model illustrates how Trichoderma spp. functions to boost plant development and control pathogens biologically

Trichoderma spp. employ several strategies to function as biocontrol agents [30]. They can rapidly multiply or utilize available food sources more efficiently than soil-borne pathogens, outcompeting them and seizing control through efficient nutrient competition. They may also engage in mycoparasitism/hyperparasitism, feeding on a pathogenic species. Additionally, Trichoderma spp. also secrete secondary metabolites that inhibit or significantly delay the growth of infectious soil-borne pathogens in their vicinity, a process known as antibiosis [25]. For example, various secondary metabolites with antimicrobial potential have been identified, such as gliotoxin from *T. lignorum*, gliovirin from *T. virens*, alamethicin F30, a peptaibol from *T. viride*, and harzianolide, an antifungal butanolide compound from *T. harzianum*. Many other such metabolites have been extensively reviewed in the report by Khan et al. (2020) [31]. The secondary metabolites secreted by Trichoderma spp. exhibit antifungal and antimicrobial effects through various mechanisms of action. These include inducing cytotoxicity by producing toxins, inhibiting spore germination, hyphal elongation, and mycelial growth, as well as suppressing the formation of sexual structures. In bacteria, these metabolites interfere with cell division and cause cell wall degradation [32]. Furthermore, Trichoderma spp. can induce plants to produce chemicals that induce localized or systemic resistance in plants. Finally, their ability to grow endophytically supports the growth of plants (Fig. 1).

## Trichoderma as a Safeguard for the Health of Plants

Trichoderma species either actively attack their hosts in their defense mechanisms or succeed by stopping the pathogen from proliferating in the host’s surroundings. They use lytic enzymes, proteolytic enzymes, ABC transporter membrane pumps, diffusible or volatile metabolites, etc. Ascomycetes, basidiomycetes, and oomycetes fungus as well as nematodes are all controlled by Trichoderma species [33]. Protease, chitinase, glucanase, tubulins, proteinase, xylanase, monooxygenase, galacturonase, cell adhesion proteins, and stress tolerance genes are a few significant categories of biocontrol genes that are readily isolated, cloned, and reported. These genes carry out particular tasks in a biocontrol mechanism, including cell wall disintegration, hyphal growth, stress tolerance, and parasite activity. The structural proteins known as tubulins, composed of microtubules, are useful for analyzing the composition of pathogen cell walls. Chitinase facilitates the hydrolysis of glycosidic linkages, while glucose oxidase catalyzes the conversion of D-glucose into D-glucono-1, 5-lactone, and hydrogen peroxide, all of which have antifungal properties. Xylanase assists in the hydrolysis of hemicellulose, which is a major component of plant cell walls [34]. Genes related to biocontrol and mycoparasitism are triggered by several signal transduction pathways, such as the cAMP pathway and mitogen-activated protein kinase (MAPK) cascades [35]. Particularly important in the heterotrimeric G protein signaling pathway is the MAP-kinase TVK1, which was identified in *T. virens* and its orthologs in *T. asperellum* (TmkA) and *T. atroviride* (TMK1) [36]. TGA1 is crucial in managing coiling around host hyphae and generating antifungal compounds. When TGA1 is missing, the growth of host fungi is significantly more hindering [37]. TGA3, on the other hand, is essential for biocontrol since strains created after the homologous gene was deleted were not pathogenic. Recently, a significant function in the biocontrol of *T. virens* has been attributed to the homolog of the VELVET protein, which is currently mostly recognized as the light-dependent regulator protein [38].

### Secondary Metabolites

The Trichoderma fungus is incredibly adept at occupying many ecological niches and its varied metabolism allows it to produce various secondary metabolites and catabolize many substrates. The secondary metabolites include about 370 distinct kinds of chemical compounds with antagonistic effects which play a crucial role in safeguarding plant health [39, 40]. The peptide antibiotic Paracelsin was one of the earliest secondary metabolites from Trichoderma spp. to be described. Trichoderma spp. produces secondary metabolites, including antifungal metabolites from some chemical com-pound classes, depending on the strain. Ghisalberti and Sivasithamparam divided them into three groups: Water-soluble substances, such as koningic or peptidic acid, volatile antibiotics, which include δ-lactone 6-pentyl-α-pyrone (6-PP) and most isocyanide derivatives [33, 41]. According to Vinale et al. (2008), peptaibols are linear oligopeptides with 12–22 amino acids that are rich in α-aminoisobutyric acid, N-acetylated at the N-terminus, and amino alcohol phenylalaninol (Pheol) or tryptophanol (Trpol) at the C-terminus [42]. The most investigated secondary metabolites are peptaibols, polyketides, pyrones, terpenes, and molecules like diketopiperazine [43] (Table 2).

**Table 2:** Secondary metabolites of certain Trichoderma isolates

Sources	Secondary metabolites	Biological task	Other applications	References
*T. hamatum, T. pseudokoningii*	Mannitol		Antimutagenic	[44]
*T. koningii*	Methyl benzoate p-hydroxy benzyl alcohol			[45]
*T. pseudokoningii*	2,5-dimethoxybenzoquinone		Cytotoxic	[45]
*T. pseudokoningii*	Succinic acid, Itaconic acid			[45]
*Trichoderma* sp.	Carolic acid			[46]
*T. viride ATCC74084*	Viridiofungin A, Viridiofungin B Viridiofungin C	Antifungal	Squalene synthase Inhibitor	[47]
*T. viride PRL 2233*	1,8-dihydroxy-3-methyl anthraquinone	Anticeptic, viricide	Cytotoxic	[48]
*T. viride PRL 2233*	1,6,8-trihydroxy-3-methyl Anthraquinone			[48]
*T. viride*	1,3,6,8 tetrahydroxy-4-aacetyl anthraquinone 1,3,6,8-tetrahydroxy Anthraquinone	Antibacterial		[49]
*T. longibrachiatum* *ATCC2449*	Sorbicillin, Bisvertinolone, Trichodimerol, Trichodermolide, Sorbiquinol		Inhibitor tumor necrosis factor	[50]
*T. harzianum IMI298371*	Harzianopyridone	Antifungal		[51]
*T. harzianum IMI 311092*	Dehydro harzianolide	Antifungal		[52]
*T. harzianum SY-307*	Harzianic acid	Hypercholesterimic		[53]
*T. harzianum*	Trichoharzin	Inhibit fungal activity, Antimicrobial activity	Regulate plant growth	[54]
*Trichoderma* spp.	Massoilactone	Antifungal activity	Regulate plant growth	[55]
*Trichoderma* spp	δ-decenolactone	Antifungal activity		[55]
*T. harzianum IMI 311090*	Koninginin E	Antifungal activity	Regulate plant growth	[56]
*T. virens ATCC74180*	3,4,14-trihydroxycarotane-14-oleate	Antifungal activity	Antitrichomal, Mycotoin	[57]
*Trichoderma. polysporum CMI40624* *T. sporulosumn CMI104643*	Trichodermin	Antifungal activity	Mycotoxin	[58]
*T. pseudokoningii* *T. hamatum HLX 1379* *HLX 1360 T. hamatum* *TK-1 T. koningii* *UC 4875 T. viride* *T. polysporum* *HLX 1379 T. hamatum* *HLX 1379 T. hamatum* *HLX 1379 T. hamatum* *HLX 1379 T. hamatum* *IMI 3208 T. hamatum*	Epifridelenol Isonitrinic acid F Dermadin Dermadin methyl ester Epoxy diol Spirolactone Antibiotic Diol isocyanide Epidiol isocyanide Isonitrin A	Antibiotic Antibiotic Antibiotic Antibiotic Antibiotic Antibiotic Antibiotic Antibiotic Antibiotic	Immunosuppressive	[55] [44]

### A Crucial Aspect of Successful Biocontrol: Mycoparasitism

Mycoparasitism is a condition in which an antagonistic fungus, known as a mycoparasite, parasitizes another fungus, referred to as the host [39]. Necrotrophic mycoparasites are the general classification for the fungus of the Trichoderma genus [59]. Around 75 Trichoderma spp. are known to have a strong propensity to become mycoparasitic [60]. Trichoderma necrotrophs physically combat fungal diseases by vigorously branching and coiling around the host’s hyphae, as well as by chemotactically attaching to the host and sensing prey. This action is known as mycoparasitism. Trichoderma can also produce pathogen appressoria homologs or penetrate using structures resembling appressoria [61, 62]. It produces antifungal compounds and hydrolytic enzymes that chemically degrade and break down the cell wall of the pathogen, eventually leading to the death of host in the final stage of the mycoparasitic interaction [59, 61]. The necrotrophic mycoparasitic effect was noted in the study by Błaszczyk et al. [63] when *T. atroviride* AN240 and *T. viride* AN255 strains interacted with *F. graminearum* and *F. avenaceum*, respectively. Similarly, it was found that the *R. solani* hyphae benefited from the mycoparasites *T. virens* [64] and *T. harzianum* [65]. Furthermore, the mycoparasitic *T. cerinum* Gur1 strain reduced chickpea wilt disease in vivo [66].

### Synthesis of Antibiotics and Additional Antifungal Substances

Most Trichoderma strains primarily produce polyketides and peptidaibols, which are volatile organic chemicals [67]. Approximately 80% of the entries in the “Peptaibiotics Database” are related to different species within the genus *Trichoderma*, making it one of the most abundant sources of peptaibols [68]. Peptaibols are categorized as antimicrobial polypeptides with a molecular weight between 500 and 2200 Da. They are rich in non-proteinogenic amino acids, especially alpha-aminoisobutyric acid and isovaline. Peptidoglycan synthesis is carried out by non-ribosomal peptide synthetases [67]. Three major non-ribosomal peptide synthetase genes, tex1, tex2, and tex3 have been recognized in the Trichoderma genomes [60]. Depending on the strain, Trichoderma spp. produce secondary metabolites that include antifungal substances from several chemical class components. The polyketides produced by these fungal species are a structurally diverse class of physiologically active chemicals found in bacteria, plants, and fungi [69]. These include pigments, mycotoxins, and antibiotics (such as macrolides and tetracyclines) [70]. Numerous Trichoderma spp. produce secondary metabolites classified as pyrones, anthraquinones, terpenoids, and epipolythiodioxopiperazines [71]. The terpenoids produced by the Trichoderma spp., include tetracyclic diterpenes (like harziandion), sesquiterpenes (like trichothecenes, like trichodermin and harzianum A), and triterpene viridian [69]. Additionally, *T. viride*, *T. harzianum*, and *T. koningii* manufacture the volatile antibiotic 6-phenyl-α-pyrone, which is responsible for the biological barrier against *F. oxysporum* having the unique coconut odour [72].

### Plant Resistance Induced by Trichoderma: the Battle for Space and Nutrients Amid Biological Stress

Pathogens can be deprived of space and nutrients when antagonistic fungi invade shared habitats such as rhizospheres, plant tissues, or phyllospheres [73]. This depends on their traits, the degree to which the host plant has colonized them, and the degree to which they have adapted to their environment [67]. Trichoderma should be common in a niche where there is a rivalry with other fungi and have effective plant colonization strategies to effectively compete with diseases for nutrients and habitats. In terms of glucose and sucrose, the Trichoderma fungus grows quite quickly [74]. Compared to other microbes, the fungus from the genus Trichoderma is far more adept in mobilizing and absorbing nutrients from the soil [75]. This procedure yields the formation of citric, gluconic, fumaric, and organic acids, which decrease the soil’s pH and encourage the solubilization of phosphates and microelements like manganese, iron, and magnesium [42]. Of particular importance in the competitive dynamics of plant–microbe and Trichoderma tripartite interaction is the production of siderophores, which is formed during an iron-deficiency stress. Siderophores are low molecular weight compound with a high affinity for iron (Fe), and help the fungi compete for iron by binding to the insoluble form (Fe^3+^) [76]. Later the insoluble Fe3 + is converted into Fe^2+^, which is readily taken up by microbes and plants [42]. Based on their chemical structure and iron coordination sites, the microbial siderophores are typically categorized into three classes, such as hydroxamate, catecholate, and carboxylate [60]. This process of iron bioavailability under stress due to siderophore production and iron solubilization ultimately triggers plant resistance by enhancing nutrient availability and strengthening the plant’s defenses against pathogens.

**Fig. 2:**
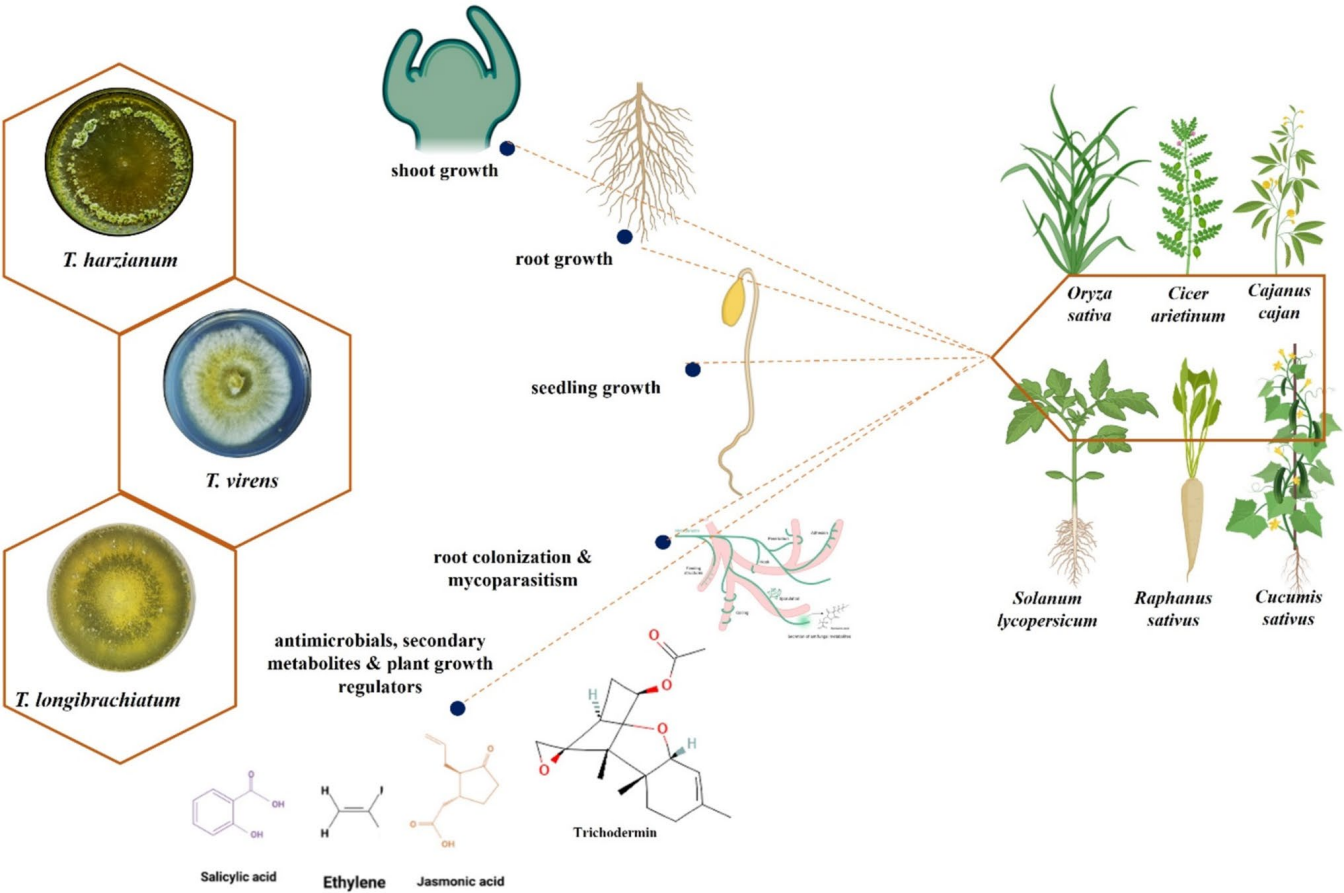
Tricoderma spp. function as biocontrol agents in various crops to improve plant growth promotion. The different Trichoderma species such as *T. harzianum*, *T. virens*, and *T. longibrachiatum* are used in different field crops and horticultural crops that promote plant growth and development by regulating shoot growth, root growth, seedling growth, root colonization, and mycoparasitism, production of antimicrobial substance such as trichodermin, trichdermamide, viridine, harzianum, etc. as well as the production of plant growth regulators such as jasmonic acid, salicylic acid, and ethylene that help in plant defense processes [Figures made using Biorender (https://app.biorender.com/signin) and adapted from [79–82]

Moreover, the Trichoderma spp. can also indirectly influence dangerous microbes through plants by triggering their systemic or local defensive mechanisms [67, 77]. Different elicitors secreted by the cells of microbes and plant tissues cause the induction of plant resistance. There are two categories for the elicitors: (1) Race-specific elicitors only cause gene-to-gene type defence in specified host cultivars (2) Nonrace-specific defence is induced in both host and non-host plants by generic elicitors delivered from pathogenic and non-pathogenic strains simultaneously. The discovery of conserved domains, such as the microbe- or pathogen-associated molecular patterns (MAMP/PAMP), is the major foundation for the plant defence response (Fig. 2) [78]. These domains activate two types of innate immunity in plants: PAMP-triggered immunity (PTI) and effector-triggered immunity (ETI) [79].

### Antibiosis

It is an antagonistic relationship between two bacteria, where the release of antibiotics or metabolites by one negatively impacts the other. According to chemical and analytical reports, 373 distinct secondary metabolites, including non-volatile and volatile terpenes, peptaibols, pyrones, and compounds containing nitrogen, were obtained from Trichoderma species and showed great potential for the production of antibiotic and secondary metabolites [11, 77]. Some of the examples that are effective against the target pathogen in situ are alkyl pyrones, trichodermin, diketopiperazines, viridin, polyketides, isonitriles, peptaibols, and sesquiterpenes isolated from Trichoderma spp., and 6-Pentyl-2H-pyran-2-one [83].

## Application of Trichoderma as a Protective Measure for Plant Health

### Trichoderma as a Bioremediation Agent

Trichoderma spp. thrives in plant roots and soil, where they combat fungal infections and demonstrate resilience against most agrochemicals. Furthermore, they exhibit high resistance to various environmental pollutants such as tannery effluents, organometallic compounds, heavy metals, and hazardous chemicals like cyanide. As a result, these fungal genera are well suited to investigate as a genetic re-source for use in the phytoremediation of harmful contaminants [84]. Trichoderma bioremediation techniques for inorganic contaminants including heavy metals and others can be categorized into four categories:

#### Biosorption

Biosorption is the ability of biological materials to extract heavy metals from wastewater via physicochemical or metabolic absorption mechanisms. It entails attaching to free groups of negatively charged molecules in a variety of biopolymers that make up the microbial cell wall in a metabolism-independent manner. For example, using the batch approach, the dried biomass of Trichoderma sp. was tested for removing harmful heavy metal ions at concentrations ranging from 0.5 to 2.0 mg/L at different pH levels [85]. The biomass of *T. harzianum* was found to significantly absorb Cr (VI) ions from the aqueous solution. FTIR spectroscopy revealed that the amine in chitin and the chitosan in the fungal cell wall play a crucial role in metal binding [86].

#### Bioaccumulation

The energetically dependent metal inflow mechanism living cells use during bioaccumulation is the active removal of the metal procedure. The ability to withstand and gather heavy metals like cadmium, zinc, copper, and arsenic in vitro have been demonstrated for various Trichoderma species. Trichoderma spp. has been shown to improve the solubility of soil micronutrients like Zn, Cu, Fe, and Mn. Cu (II) binding in the cell wall surface was demonstrated by *T. viride* as a metal tolerance mechanism. At pH 5.0 and 100 mg/L of Cu (II) at 30 °C, a maximum of 80% of the copper was eliminated in 72 h. Copper was rendered less dispersed and accessible in the media by binding to the cell surface, which decreased the metal’s toxicity [87].

#### Biovolatilization

It involves the enzymatic conversion of organic and inorganic metalloid compounds into their volatile by-products, a process known as biomethylation. *T. viridian* and *T. asperellum* have been shown to be arsenic in liquid surroundings and should be volatilized. The fungal strains Rhizopus sp., Neocosmospora sp., Trichoderma sp., and sterile mycelial strain were found to have the most effective at removing arsenic from soil [88].

#### Phytoremediation

Microbe-assisted utilizing plants and microbes to remove toxins biologically are called phytoremediation, also known as phytobial remediation. Trichoderma spp. aids in phyto extraction processes that promote the absorption of other ions, including nitrates, in the root area and adopt certain hazardous metals and metalloids. *Pteris vittata*, an arsenic-accumulating fern, grows more roots when *T. harzianum* strains are used because they can detoxify potassium cyanide (Table 3).

**Table 3:** Trichoderma spp.–based bioremediation of various pollutants

Sl no	Pollutants	Trichoderma spp.	References
1	A solvent that is organic	Trichoderma spp.	[89]
2	Dichlorvos, an organophosphate insecticide	*T. atroviride*	[90]
3	Phenanthrene, Crude oil, naphthalene, and benzopyrene	*11 Trichoderma* strains	[91]
4	Tolerance to arsenic in *Eucalyptus* globules	*T. harzianum*	[92]
5	Cd and Ni-contaminated soils for phytoextraction	*T. atroviride*	[93]
6	Eucalyptus’s resistance to aluminium	*T. harzianum*	[94]
7	Metal-contaminated soil using PGPR	*T. harzianum*	[95]
8	Polyresistance pesticide	Trichoderma spp.	[96]
9	Cyanide	Trichoderma spp.	[97]
10	Contaminants in the soil and water	Trichoderma spp.	[98]
11	Diesel-contaminated soil	Trichoderma	[99]
12	Agrochemicals like dieldrin, penta-chloro-nitrobenzene, DDT, endosulfan, and penta-chloro-phenol)	*T. harzianum*	[100]

## The Implications of Trichoderma in Agriculture

Biological control is a method of reducing crop pests by employing helpful microbial organisms. Among the many beneficial bacteria, Trichoderma sp. is frequently utilized as a biocontrol agent against various plant diseases. Trichoderma sp. is active rhizosphere colonizers that also infiltrate cortex cells in roots and live as endophytes. Examples include *Trichoderma harzianum*, *T. longibrachiatum*, *T. virens*, and *T. asperellum*, etc. These species contribute significantly to plant growth and metabolism, promoting increased shoot and root lengths, enhanced overall plant growth, and improved vigor and emergence seedling in many crops such as beans, brinjals, cauliflower, chickpeas, cucumbers, lentils, pigeon peas, radishes, tomatoes, and rice. Figure 2 provides a schematic representation illustrating the plant growth-promoting activities of Trichoderma species. Trichoderma antibiotics such as viridin, gliotoxin, enzymatic breakdown of cell walls, and physiologically active heat-stable metabolites like ethyl acetate are engaged in preventing illness and promoting plant development. Examples of important categories of biocontrol genes include xylanase, chitinase, tubulins, protease, glucanase, proteinase, galacturonase, genes encoding cell adhesion proteins, monooxygenase, and stress tolerance genes that are easily isolated, cloned, and described. These genes carry out particular tasks within the biocontrol mechanism, including cell wall disintegration, hyphal growth, stress tolerance, and parasite activity. Tubulins are microtubule-derived structural proteins that facilitate the examination of the content of pathogen cell walls. Chitinase facilitates the hydrolysis of glycosidic bonds. D-glucose is converted by glucose oxidase into hydrogen peroxide, 5-lactone, and D-glucono-15-lactone, all of which have antifungal qualities. One important component of plant cell walls, hemicellulose, is broken down with the help of xylanase [101].

### Effectiveness of Trichoderma Species Against Fungus that are Found in the Soil

Trichoderma spp. is used as plant growth enhancers and antagonistic fungal agents against various pests. Faster metabolic rates, antimicrobial metabolites, and physio-logical conformation are important elements that primarily lead to these fungi's antagonistic interactions. It is commonly recognized that Trichoderma fungi are antagonistic to several bacteria, invertebrates, and other soil-phytopathogens [19]. The efficiency of Trichoderma species against soil-dwelling fungi is shown in (Table 4).

**Table 4:** Effectiveness of Trichoderma species against fungus that are found in the soil

Trichoderma strains	Pathogens	Plant/crops	Disease with efficacy	References
*TH-3 T. harzianum*	*Rhizoctonia solani*	Tomato	Wilt (5%)	[102]
*T. harzianum*	*Fusarium solani*	Tomato	Root rot (70–72%)	[103]
*T. harzianum*	*R. solani*	Tomato	Damping off (51%)	[103]
*T. harzianum* *Mutants*	*R. solani*	Tomato	Damping off (40%)	[103]
*T. viride (Tv-R)* *T. harzianum*	*M. phaseolina* *R. bataticola*	Chickpea Mungbean	Root rot (62%) Dry root (87%) rot	[104]
*T. harzianum* *T-22*	*F. verticillioide*	Maize	Ear and kernel Rot (65% reduce size of necrotic area )	[105]
*T. viride*	*F. oxysporum* *f. sp. Adzuki*	Soybean	Root rot	[106]
*T. harzianum*	*Cucumerinum*	Cucumber	Stem and root (12–79%) Rot	[107]
*T. harzianum*	*Alternaria* *tenuissima*	Sorrel	Leaf spot (67–76%)	[107]
*T. viride*	*F. verticilloid A. alternate*	Tomato	Root rot and wilt 67%	[107]
*T. harzianum* *T. longibrachiatum*	*A. porri*	Onion	Purple blotch 73%	[108]
*T. viride*	*Colletotrichumcapsici*	Chilli	Fruit rot 58%	[109]
*T. harzianum*	*F. udum*	Pigeon pea	Wilt (*T. harzianum* is more successful when applied to the soil than when seed is used)	[110]
*T. hamatum*	*F. oxysporum*	Lentil	Vascular wilt (33%)	[111]

#### Timber Preservation

Ejechi studied *T. viride*’s capacity to prevent *G. sepiarium* and *Gloeophyllum* sp. from decomposing obeche (Triplochiton sceleroxylon) wood over an 11-month period, during the wet and dry seasons in tropical climate. *T. viride* successfully suppressed the decay fungus through mycoparasitism and nutritional competition, [112]. Trichoderma isolates can inhibit and kill wood decay fungi by the release of volatile organic compounds, with production varying based on the specific Trichoderma isolate [113]. Trichoderma fungi are found on freshly cut sawn wood of many different softwood and hardwood species as well as in soils across all latitudes. The fungi that develop on wood surfaces have the potential to reduce the value of sawn objects by reducing the functional aesthetics of lignocellulosic materials. Under favorable conditions, the Trichoderma fungi can even cause soft rot in wooden materials because they produce a wide variety of enzymes, including cellulase, hemi-cellulase, xylanase, and chitinase [113]. Overall, the diverse capabilities of Trichoderma fungi highlight their significant impact on both wood preservation and degradation processes.

#### Tolerance to Abiotic and Biotic Stresses

Trichoderma species, an excellent natural protein source, can help plants with-stand biotic and abiotic stress conditions. It has also been reported that the cloned and characterized hsp70 gene from *T. harzianum* T34 isolate encodes a protein that, when expressed and produced in Arabidopsis, increases tolerance to heat and other abiotic stimuli. This gene codes for a protein product that allows the fungus to resist heat and other stresses, such as oxidative, salt, and osmotic tolerances, to reach higher levels. With the recent sequencing of the genomes of seven Trichoderma species, there is promising potential for developing transgenic plants that may provide effective resistance to changing climate conditions [114].

#### Reaction Sensitivity to Agrochemicals

The efficacy of the bioagents is decreased by the toxic character of the fungicides used in crop production technologies. Consequently, researchers have investigated Trichoderma sensitivity and tolerance [115]. Studies have been conducted on the impact of different fungicides in combination with Trichoderma species on treating illnesses holistically. Trichoderma spp. has been proven to be more resistant to broad-range fungicides than many other soil microbes because of their capacity to colonize pesticide-treated soil more quickly [116]. Because numerous unusual contaminants can be treated simultaneously and have a wider range of uses, trichomoniasis alone, in conjunction with bacteria or immobilized formulations, can show enormous potential. This will boost the overall cost-effectiveness of the method.

### Future Prospects

Numerous studies have shown that Trichoderma spp. can tolerate and detoxify environmental contaminants from contaminated areas. The synthesis of amylases from *T. harzianum*, cellulases from *T. reesei*, 1,3 β-glucanases from *T. harzianum*, *T. koningii*, and chitinases from *T. aureoviridae* and *T. harzianum* is well known. The genus Trichoderma is a good source of various hydrolytic and industrially important enzymes. They have been used in the manufacture of extracellular gold nanoparticles using *T. koningii* and the fabrication of silver nanoparticles (AgNPs) using *T. reesei* [104]. However, further investigation is needed to examine the long-term consequences on the stability and rehabilitation of the contaminated sites before these interactions between Trichoderma and plants can be fully exploited. The survival of Trichoderma depends on the diverse metabolic capabilities of this group of fungi. A deeper understanding of these processes will lead to better, more affordable environmental protection techniques and increased crop output in contaminated areas. Due to their broad spectrum of biotic and abiotic stress tolerance, Trichoderma species have the potential to be exploited in sustainable agriculture and biofuel crops with the help of modern plant biology methods and techniques.

## Conclusion

The overuse of chemical fertilizers and pesticides has negatively impacted human health and the environment. Consequently, research on organisms as biocontrol agents has emerged as a promising approach to finding sustainable and eco-friendly alternatives. Trichoderma**,** due to its multiple biocontrol traits, is one of the most extensively studied beneficial microbes for managing various plant pathogens. These are free-living soil fungi that colonize decomposing organic matter and form beneficial endophytic associations with plants. They inhibit phytopathogenic fungi while stimulating plant defenses, promoting root development, and enhancing plant growth under biotic and abiotic stresses. It is effective not only against fungi and oomycetes but also against insects, pests, and nematodes. This is achieved through enhanced plant defenses or by directly inhibiting pathogen growth via competition, antibiosis, or parasitism. However, a deeper understanding of these underlying processes can significantly improve the effectiveness of Trichoderma in managing plant-pathogen interactions. This would make Trichoderma a highly effective biocontrol agent, biofungicides, and biofertilizers thereby reducing dependence on synthetic chemicals and promoting sustainable agriculture. Although numerous formulations containing different Trichoderma species are available for sustainable crop production, their high cost often limits accessibility for small-scale farmers. Species like *T. atroviride* and *T. harzianum* are notable mycoparasites, while newly discovered strains hold promise as cost-effective alternatives for farmers. Additionally, the production of hydrolytic enzymes and N-acetylglucosamine (GlcNAc) from Trichoderma spp, which influences signaling and virulence properties in bacteria, highlights its diverse biocontrol functions. Despite their potential, broader accessibility and cost reduction are needed to maximize their application in sustainable crop production.

## Data Availability

No datasets were generated or analysed during the current study.

## References

[1] Singh A, Shukla N, Kabadwal BC, Tewari AK, Kumar J (2018) Review on plant-Trichoderma-pathogen interaction. Int J Curr Microbiol Appl Sci 7(2):2382–2397. 10.20546/ijcmas.2018.702.274

[2] Savary S, Ficke A, Aubertot JN, Hollier C (2012) Crop losses due to diseases and their implications for global food production losses and food security. Food Security 4(4):519–537. 10.1007/s12571-012-0201-1

[3] Sharma A, Sharma NK, Kardile HB (2021) Soil borne fungal diseases and their control in below ground crops. In Microbial Biotechnology in Crop Protection (pp 251–268). Singapore: Springer Singapore. 10.1007/978-981-15-5905-1_12

[4] De A, Bose R, Kumar A, Subho M (2014) The targeted delivery of pesticides using biodegradable polymeric nanoparticles. Springer India. 10.1007/978-81-322-1764-4

[5] Shattuck A, Werner M, Mempel F, Dunivin Z, Galt R (2023) Global pesticide use and trade database (GloPUT): new estimates show pesticide use trends in low-income countries substantially underestimated. Global Environ Change 81:102693. 10.1016/j.gloenvcha.2023.102693

[6] Ngegba PM, Cui G, Khalid MZ, Zhong G (2022) Use of botanical pesticides in agriculture as an alternative to synthetic pesticides. Agriculture 12(5):600. 10.3390/agriculture12050600

[7] Ghazanfar MU, Raza M, Raza W, Qamar MI (2018) Trichoderma as potential biocontrol agent, its exploitation in agriculture: a review. Plant Protect 2(3). 10.18193/plant-protection-2018-2-3

[8] Muthukumar A, Udhayakumar R, Naveenkumar R (2016) Eco friendly management of damping-off of solanaceous crops caused by Pythium species. In Current Trends in Plant Disease Diagnostics and Management Practices (pp. 49–90). Springer, Cham. 10.1007/978-3-319-32396-2_4

[9] Singh R, Tomer A, Prasad D, Viswanath HS (2020) Biodiversity of Trichoderma species. 10.1007/978-981-15-2678-7_3

[10] Keswani C, Mishra S, Sarma BK, Singh SP, Singh HB (2014) Unraveling the efficient applications of secondary metabolites of various Trichoderma spp. Appl Microbiol Biotechnol 98(2):533–544. 10.1007/s00253-013-5414-7 24276619 10.1007/s00253-013-5344-5

[11] Contreras-Cornejo HA, Macías-Rodríguez L, Del-Val EK, Larsen J (2016) Ecological functions of Trichoderma spp. and their secondary metabolites in the rhizosphere: interactions with plants. FEMS Microbiol Ecol 92(4):36. 10.1093/femsec/fiw036 10.1093/femsec/fiw036 26906097

[12] Bissett J (1991) A revision of the genus Trichoderma. II. Infrageneric classification. Can J Bot 69(11):2357–2372. 10.1139/b91-282

[13] Dini I, Alborino V, Lanzuise S, Lombardi N, Marra R, Balestrieri A, Ritieni A, Woo SL, Vinale F (2022) Trichoderma enzymes for degradation of Aflatoxin B1 and Ochratoxin A. Molecules 27(12):3959. 10.3390/molecules27123959 35745082 10.3390/molecules27123959 PMC9231114

[14] Samuels GJ (2006) Trichoderma: systematics, the sexual state, and ecology. Phytopathology 96(2):195–206. 10.1094/PHYTO-96-0195 18943925 10.1094/PHYTO-96-0195

[15] Kulkarni S, Sagar SD (2007) Trichoderma–a potential biofungicide of the millennium. Bulletin published by the Dept. of Plant Pathology, UAS, Dharwad, pp 1–20

[16] Calpas JT (2005) Development of a biological control for botrytis stem canker of greenhouse tomatoes caused by Botrytis cinerea Pers (pp. 5482–5482). Library and Archives Canada= Bibliothèque et Archives Canada, Ottawa

[17] Ghazanfar MU, Raza M, Raza W, Qamar MI (2018) Trichoderma as potential biocontrol agent, its exploitation in agriculture: a review. Plant Prot 2(3). 10.21608/ppj.2018.3148

[18] Kumar V, Verma DK, Pandey AK, Srivastava S (2019) Trichoderma spp.: identification and characterization for pathogenic control and its potential application. In Microbiology for Sustainable Agriculture, Soil Health, and Environmental Protection (pp 223–258). Apple Academic Press. 10.1201/9780429288478

[19] Schuster A, Schmoll M (2010) Biology and biotechnology of Trichoderma. Appl Microbiol Biotechnol 87(3):787–799. 10.1007/s00253-010-2576-8 20461510 10.1007/s00253-010-2632-1 PMC2886115

[20] Changela D, Ramani K, Dangar K, Vachhani K, Raval M, Kalasava A (2022) Mining the potential and biodiversity of Trichoderma in the domain of agriculture. In Beneficial Microorganisms in Agriculture (pp 211–229). Springer, Singapore. 10.1007/978-981-16-5181-1_13

[21] Abirami S, Gayathri SS, Usha C (2022) Trichoderma as biostimulant-a plausible approach to alleviate abiotic stress for intensive production practices. In New and Future Developments in Microbial Biotechnology and Bioengineering (pp 57–84). Elsevier. 10.1016/B978-0-323-91724-6.00005-8

[22] Zhang CL, Druzhinina IS, Kubicek CP, Xu T (2005) Trichoderma biodiversity in China: evidence for a North to South distribution of species in East Asia. FEMS Microbiol Lett 251(2):251–257. 10.1016/j.femsle.2005.08.008 16165315 10.1016/j.femsle.2005.08.034

[23] Al-Ani LKT (2018) Trichoderma from extreme environments: physiology, diversity, and antagonistic activity. In Extremophiles in Eurasian Ecosystems: Ecology, Diversity, and Applications (pp 389–403). Springer, Singapore. 10.1007/978-981-10-8134-3_19

[24] Chaverri P, Branco-Rocha F, Jaklitsch W, Gazis R, Degenkolb T, Samuels GJ (2015) Systematics of the Trichoderma harzianum species complex and the re-identification of commercial biocontrol strains. Mycologia 107(3):558–590. 10.3852/14-147 25661720 10.3852/14-147 PMC4885665

[25] Kariuki C (2020) Evaluation of Bacillus and Trichoderma species for biological control of bacterial wilt caused by Ralstonia solanacearum in tomato (Doctoral dissertation, University of Nairobi). 10.13140/RG.2.2.29100.80001

[26] Ru Z, Di W (2012) Trichoderma spp. from rhizosphere soil and their antagonism against Fusarium sambucinum. Afr J Biotechnol 11(18):4180–4186. 10.5897/AJB12.2167

[27] Sekhar YC, Ahammed SK, Prasad TNVKV (2017) Identification of Trichoderma species based on morphological characters isolated from rhizosphere of groundnut (*Arachis hypogaea* L). Int J Sci Environ Technol 6(3):2056–2063

[28] Vipul K, Mohammad S, Muksesh S, Sonika P, Anuradha S, Sharma A (2014) Role of secondary metabolites produced by commercial Trichoderma species and their effect against soil borne pathogens. Biosens J 3(2). 10.1155/2014/781989

[29] Williams J, Clarkson JM, Mills PR, Cooper RM (2003) A selective medium for quantitative reisolation of Trichoderma harzianum from Agaricus bisporus compost. Appl Environ Microbiol 69(7):4190–4191. 10.1128/AEM.69.7.4190-4191.2003 12839798 10.1128/AEM.69.7.4190-4191.2003 PMC165142

[30] Rajesh RW, Rahul MS, Ambalal NS (2016) Trichoderma: a significant fungus for agriculture and environment. Afr J Agric Res 11(22):1952–1965. 10.5897/AJAR2016.11359

[31] Khan RAA, Najeeb S, Hussain S, Xie B, Li Y (2020) Bioactive secondary metabolites from Trichoderma spp. against phytopathogenic fungi. Microorganisms 8(6):817 32486107 10.3390/microorganisms8060817 PMC7356054

[32] Al-Ani LKT (2019) Bioactive secondary metabolites of trichoderma spp. for efficient management of phytopathogens. In: Singh H, Keswani C, Reddy M, Sansinenea E, García-Estrada C (eds) Secondary metabolites of plant growth promoting rhizomicroorganisms. Springer, Singapore. 10.1007/978-981-13-5862-3_7

[33] Schuster A, Schmoll M (2010) Biology and biotechnology of Trichoderma. Appl Microbiol Biotechnol 87(3):787–799. 10.1007/s00253-010-2570-8 20461510 10.1007/s00253-010-2632-1 PMC2886115

[34] Sharma P, Kumar V, Ramesh R, Saravanan K, Deep S, Sharma M, … Dinesh S (2011) Biocontrol genes from Trichoderma species: a review. Afr J Biotechnol 10(86):19898–19907. 10.5897/AJB11.2831

[35] Zeilinger S, Omann M (2007) Trichoderma biocontrol: signal transduction pathways involved in host sensing and mycoparasitism. Gene Regul Syste Biol 1:GRSB-S397. 10.4137/GRSB.S397 10.4137/grsb.s397 PMC2759141 19936091

[36] Mendoza-Mendoza A, Rosales-Saavedra T, Cortes C, Castellanos-Juarez V, Martínez P, Herrera-Estrella A (2007) The MAP kinase TVK1 regulates conidiation, hydrophobicity and the expression of genes encoding cell wall proteins in the fungus Trichoderma virens. Microbiology 153(7):2137–2147. 10.1099/mic.0.2006/003326-0 17600058 10.1099/mic.0.2006/005462-0

[37] Reithner B, Brunner K, Schuhmacher R, Peissl I, Seidl V, Krska R, Zeilinger S (2005) The G protein α subunit Tga1 of Trichoderma atroviride is involved in chitinase formation and differential production of antifungal metabolites. Fungal Genet Biol 42(9):749–760. 10.1016/j.fgb.2005.06.001 15964222 10.1016/j.fgb.2005.04.009

[38] Mukherjee PK, Kenerley CM (2010) Regulation of morphogenesis and biocontrol properties in Trichoderma virens by a VELVET protein, Vel1. Appl Environ Microbiol 76(7):2345–2352. 10.1128/AEM.02653-09 20154111 10.1128/AEM.02391-09 PMC2849264

[39] Ghorbanpour M, Omidvari M, Abbaszadeh-Dahaji P, Omidvar R, Kariman K (2018) Mechanisms underlying the protective effects of beneficial fungi against plant diseases. Biol Control 117:147–157. 10.1016/j.biocontrol.2017.11.007

[40] Eslahi N, Kowsari M, Zamani MR, Motallebi M (2022) Correlation study between biochemical and molecular pathways of Trichoderma harzianum recombinant strains on plant growth and health. J Plant Growth Regul 41(4):1561–1577. 10.1007/s00344-021-10423-6

[41] Saldaña-Mendoza SA, Pacios-Michelena S, Palacios-Ponce AS, Chávez-González ML, Aguilar CN (2023) Trichoderma as a biological control agent: mechanisms of action, benefits for crops and development of formulations. World J Microbiol Biotechnol 39(10):269. 10.1007/s11274-023-05618-4 37532771 10.1007/s11274-023-03695-0

[42] Vinale F, Sivasithamparam K, Ghisalberti EL, Marra R, Woo SL, Lorito M (2008) Trichoderma–plant–pathogen interactions. Soil Biol Biochem 40(1):1–10. 10.1016/j.soilbio.2007.08.012

[43] Singh HB, Singh BN, Singh S, Sarma B (2013) Exploring different avenues of Trichoderma as a potent bio-fungicidal and plant growth promoting candidate-an overview. Ann Rev Plant Pathol 5:315–321. 10.1146/annurev-phyto-082712-102503

[44] Hussain SA, Noorani R, Qureshi IH (1975) Microbial chemistry Part I. Isolation and characterization of gliotoxin, ergosterol, palmitic acid and mannitol-metabolic products of Trichoderma hamatum Bainier. J Appl Microbiol 28(1):37–42. 10.1111/j.1365-2672.1975.tb02013.x

[45] Kamal A, Akhtar R, Qureshi AA (1971) Biochemistry of microorganisms. XX. 2,5-Dimethoxybenzoquinone, tartronic acid, itaconic acid, succinic acid, pyrocalciferol, epifriedelinol, lanosta-7,9(11), 24-triene-3ß,21-diol, trichodermene A, methyl 2,4,6-octatriene and cordycepic acid, Trichoderma metabolites. Pak J Sci Ind Res 14:71–78. 10.1007/BF03216509

[46] Turner WB, Aldridge DC (1983) *Fungal Metabolites II*. Academic Press, London. Visconti di Modrone, G. (1968). Chemische und biogenetische untersuchungen eineger metaboliten von *Fusidium coccineum*. Diss. ETH 4156. Eidgenössische Technische Hochschule, Zurich. 10.1016/B978-0-12-162502-0.50011-3

[47] Harris GH, Jones ETT, Meinz MS, Nallin-Omstead M, Helms GL, Bills GF, Zink D, Wilson KE (1993) Isolation and structure elucidation of viridiofungins A, B and C. Tetrahedron Lett 34:5235–5238. 10.1016/S0040-4039(00)81232-4

[48] Slater GP, Haskins RH, Hagge LR, Nesbitt LR (1967) Metabolic products from a *Trichoderma viride* Pers. ex Fries. Can J Chem 45:92–96. 10.1139/v67-017

[49] Betina V, Kužela Š (1987) Uncoupling effect of fungal hydroxyanthraquinones on mitochondrial oxidative phosphorylation. Chem Biol Interact 62(2):179–189. 10.1016/0009-2797(87)90019-0 3594640 10.1016/0009-2797(87)90089-5

[50] Andrade R, Ayer WA, Trifonov LS (1996) The metabolites of *Trichoderma**longibrachiatum*. Part II The structures of trichodermolide and sorbiquinol. Can J Chem 74(3):371–379. 10.1139/v96-042

[51] Dickinson JM, Hanson JR, Hitchcock PB, Claydon N (1989) Structure and biosynthesis of harzianopyridone, an antifungal metabolite of *Trichoderma harzianum*. J Chem Soc Perkin Trans 1(11):1885–1887. 10.1039/P19890001885

[52] Almassi F, Ghisalberti EL, Narbey MJ, Sivasithamparam K (1991) New antibiotics from strains of *Trichoderma harzianum*. J Nat Prod 54(2):396–402. 10.1021/np50059a013

[53] Sawa R, Mori Y, Iinuma H, Naganawa H, Hamada M, Yoshida S, …, Takeuchi T (1994) Harzianic acid, a new antimicrobial antibiotic from a fungus. J Antibiot 47(6):731–732. 10.7164/antibiotics.47.731 10.7164/antibiotics.47.731 8040080

[54] Kobayashi M, Uehara H, Matsunami K, Aoki S, Kitagawa I (1993) Trichoharzin, a new polyketide produced by the imperfect fungus *Trichoderma harzianum* separated from the marine sponge *Micale cecilia*. Tetrahedron Lett 34(49):7925–7928. 10.1016/S0040-4039(00)86025-0

[55] Hill RA, Cutler HG, Parker SR (1995) Trichoderma and metabolites as control agents for microbial plant diseases. PCT Int Appl 9520879(10). 10.1016/S0045-3069(01)00499-2

[56] Ghisalberti EL, Rowland CY (1993) Antifungal metabolites from *Trichoderma harzianum*. J Nat Prod 56(10):1799–1804. 10.1021/np50082a023 8277317 10.1021/np50100a020

[57] Lee SH, Hensens OD, Helms GL, Liesch JM, Zink DL, Giacobbe RA, …, Kaczorowski GJ (1995) L-735,334, a novel sesquiterpenoid potassium channel-agonist from *Trichoderma virens*. J Nat Prod 58(12):1822–1828. 10.1021/np50128a017 10.1021/np50126a004 8691204

[58] Adams PM, Hanson JR (1972) Sesquiterpenoid metabolites of *Trichoderma**polysporum* and *T.**sporulosum*. Phytochemistry 11(1):423. 10.1016/S0031-9422(00)81795-8

[59] Seidl-Seiboth V, Ihrmark K, Druzhinina I, Karlsson M (2014) Molecular evolution of *Trichoderma* chitinases. In *Biotechnology and Biology of Trichoderma* (pp 67–78). Elsevier. 10.1016/B978-0-444-63426-8.00005-5

[60] Druzhinina IS, Seidl-Seiboth V, Herrera-Estrella A, Horwitz BA, Kenerley CM, Monte E, …, Kubicek CP (2011) *Trichoderma*: the genomics of opportunistic success. Nat Rev Microbiol 9(10):749–759. 10.1038/nrmicro2654 10.1038/nrmicro2637 21921934

[61] Mukherjee M, Mukherjee PK, Horwitz BA, Zachow C, Berg G, Zeilinger S (2012) Trichoderma–plant–pathogen interactions: advances in genetics of biological control. Indian J Microbiol 52:522–529. 10.1007/s12088-012-0245-5 24293705 10.1007/s12088-012-0308-5 PMC3516657

[62] Moreno-Ruiz D, Lichius A, Turrà D, Di Pietro A, Zeilinger S (2020) Chemotropism assays for plant symbiosis and mycoparasitism related compound screening in *Trichoderma atroviride*. Front Microbiol 11:601251. 10.3389/fmicb.2020.601251 33329491 10.3389/fmicb.2020.601251 PMC7729004

[63] Błaszczyk L, Basińska-Barczak A, Ćwiek-Kupczyńska H, Gromadzka K, Popiel D, Stępień Ł (2017) Suppressive effect of *Trichoderma* spp. on toxigenic species. Pol J Microbiol 66(1):85–100. 10.21307/pjm-2017-010 29359702 10.5604/17331331.1234996

[64] Mukherjee PK (2011) Genomics of biological control-whole genome sequencing of two mycoparasitic *Trichoderma* spp. Curr Sci 101(3):309–310. 10.18520/cs/v101/i3/309-310

[65] Almeida FBDR, Cerqueira FM, Silva RDN, Ulhoa CJ, Lima AL (2007) Mycoparasitism studies of *Trichoderma harzianum* strains against *Rhizoctonia solani*: evaluation of coiling and hydrolytic enzyme production. Biotech Lett 29:1189–1193. 10.1007/s10529-007-9335-2 10.1007/s10529-007-9372-z 17534583

[66] Khare E, Kumar S, Kim K (2018) Role of peptaibols and lytic enzymes of *Trichoderma cerinum* Gur1 in biocontrol of *Fusarium oxysporum* and chickpea wilt. Environ Sustain 1:39–47. 10.1007/s42398-018-0006-8

[67] Sood M, Kapoor D, Kumar V, Sheteiwy MS, Ramakrishnan M, Landi M, …, Sharma A (2020) Trichoderma: the “secrets” of a multitalented biocontrol agent. Plants 9(6):762. 10.3390/plants9060762 10.3390/plants9060762 PMC7355703 32570799

[68] Neumann NK, Stoppacher N, Zeilinger S, Degenkolb T, Brückner H, Schuhmacher R (2015) The peptaibiotics database–a comprehensive online resource. Chem Biodivers 12(5):743–751. 10.1002/cbdv.201500026 26010663 10.1002/cbdv.201400393

[69] Zeilinger S, Gruber S, Bansal R, Mukherjee PK (2016) Secondary metabolism in *Trichoderma*—chemistry meets genomics. Fungal Biol Rev 30:74–90. 10.1016/j.fbr.2016.04.001

[70] Delgado-Jarana J, Rincon AM, Benítez T (2002) Aspartyl protease from *Trichoderma harzianum* CECT 2413: cloning and characterization. Microbiology 148(5):1305–1315. 10.1099/00221287-148-5-1305 11988504 10.1099/00221287-148-5-1305

[71] Siddiquee S (2014) Recent advancements on the role and analysis of volatile compounds (VOCs) from Trichoderma. In: Schmoll M, Dattenböck C (Eds.), Biotechnology and Biology of Trichoderma (pp 139–175). Elsevier. 10.1016/B978-0-444-59508-2.00010-4

[72] Blaszczyk LMSKS, Siwulski M, Sobieralski K, Lisiecka J, Jedryczka M (2014) Trichoderma spp.–application and prospects for use in organic farming and industry. J Plant Protect Res 54(4):205–210. 10.2478/jppr-2014-0031

[73] Ghorbanpour M, Omidvari M, Abbaszadeh-Dahaji P, Omidvar R, Kariman K (2018) Mechanisms underlying the protective effects of beneficial fungi against plant diseases. Biol Control 117:147–157. 10.1016/j.biocontrol.2017.11.008

[74] Jaroszuk-Ściseł J, Tyśkiewicz R, Nowak A, Ozimek E, Majewska M, Hanaka A et al (2019) Phytohormones (auxin, gibberellin) and ACC deaminase in vitro synthesized by the mycoparasitic *Trichoderma* DEMTkZ3A0 strain and changes in the level of auxin and plant resistance markers in wheat seedlings inoculated with this strain conidia. Int J Mol Sci 20(19):4923. 10.3390/ijms20194923 31590281 10.3390/ijms20194923 PMC6801869

[75] Gajera H, Domadiya R, Patel S, Kapopara M, Golakiya B (2013) Molecular mechanism of *Trichoderma* as bio-control agents against phytopathogen system–a review. Curr Res Microbiol Biotechnol 1(4):133–142. 10.5958/j.2319-7625.1.4.017

[76] Khan RAA, Najeeb S, Hussain S, Xie B, Li Y (2020) Bioactive secondary metabolites from Trichoderma spp. against phytopathogenic fungi. Microorganisms 8(6):817. 10.3390/microorganisms8060817 32486107 10.3390/microorganisms8060817 PMC7356054

[77] Ghosh S, Bhagwat T, Webster TJ (2021) Endophytic microbiomes and their plant growth-promoting attributes for plant health. In: Kumar V, Sharma S (Eds.) Current Trends in Microbial Biotechnology for Sustainable Agriculture (pp. 245–278). Springer. 10.1007/978-3-030-54927-2_10

[78] Göhre V, Jones AM, Sklenář J, Robatzek S, Weber AP (2012) Molecular crosstalk between PAMP-triggered immunity and photosynthesis. Mol Plant Microbe Interact 25(8):1083–1092 22550958 10.1094/MPMI-11-11-0301

[79] Guzmán-Guzmán P, Kumar A, de Los Santos-Villalobos S, Parra-Cota FI, Orozco-Mosqueda MDC, Fadiji AE, …, Santoyo G (2023) Trichoderma species: our best fungal allies in the biocontrol of plant diseases—a review. Plants 12(3):432 10.3390/plants12030432 PMC9921048 36771517

[80] Febri Doni FD, Anizan Isahak AI, Che Radziah CMZ, Wan Mohtar WY (2014) Physiological and growth response of rice plants (Oryza sativa L.) to Trichoderma spp. inoculants 10.1186/s13568-014-0045-8 PMC4052627 24949276

[81] Prismantoro D, Akbari SI, Permadi N, Dey U, Anhar A, Miranti M, .…, Doni F (2024) The multifaceted roles of Trichoderma in managing rice diseases for enhanced productivity and sustainability. J Agric Food Res 18:101324

[82] Sharma KK (2018) Trichoderma in agriculture: an overview of global scenario on research and its application. Int J Curr Microbiol App Sci 7:1922–1933

[83] Tripathi P, Singh PC, Mishra A, Chauhan PS, Dwivedi S, Bais RT, Tripathi RD (2013) Trichoderma: a potential bioremediator for environmental clean-up. Clean Technol Environ Policy 15(4):541–550

[84] Rahman NNNA, Shahadat M, Omar FM, Chew AW, Kadir MOA (2016) Dry Trichoderma biomass: biosorption behavior for the treatment of toxic heavy metal ions. Desalin Water Treat 57(28):13106–13112

[85] Shoaib A, Aslam N, Aslam N (2013) Trichoderma harzianum: adsorption, desorption, isotherm and FTIR studies, pp 1460–1465

[86] Anand P, Isar J, Saran S, Saxena RK (2006) Bioaccumulation of copper by Trichoderma viride. Biores Technol 97(8):1018–1025 10.1016/j.biortech.2005.04.046 16324839

[87] Srivastava PK, Vaish A, Dwivedi S, Chakrabarty D, Singh N, Tripathi RD (2011) Biological removal of arsenic pollution by soil fungi. Sci Total Environ 409(12):2430–2442. 10.1016/j.scitotenv.2011.03.002 21459413 10.1016/j.scitotenv.2011.03.002

[88] Rajani P, Rajasekaran C, Vasanthakumari MM, Olsson SB, Ravikanth G, Shaanker RU (2021) Inhibition of plant pathogenic fungi by endophytic Trichoderma spp. through mycoparasitism and volatile organic compounds. Microbiol Res 242:126595. 10.1016/j.micres.2020.126595 33017769 10.1016/j.micres.2020.126595

[89] Tang J, Liu L, Hu S, Chen Y, Chen J (2009) Improved degradation of organophosphate dichlorvos by *Trichoderma atroviride* transformants generated by restriction enzyme-mediated integration (REMI). Biores Technol 100(1):480–483. 10.1016/j.biortech.2008.05.022 10.1016/j.biortech.2008.05.022 18585910

[90] Argumedo-Delira R, Alarcón A, Ferrera-Cerrato R, Almaraz JJ, Peña-Cabriales JJ (2012) Tolerance and growth of 11 *Trichoderma* strains to crude oil, naphthalene, phenanthrene, and benzo [a] pyrene. J Environ Manage 95:S291–S299 20869805 10.1016/j.jenvman.2010.08.011

[91] Arriagada C, Aranda E, Sampedro I, Garcia-Romera I, Ocampo JA (2009) Contribution of the saprobic fungi Trametes versicolor and Trichoderma harzianum and the arbuscular mycorrhizal fungi Glomus deserticola and G. claroideum to arsenic tolerance of Eucalyptus globulus. Bioresour Technol 100(24):6250–6257 19648001 10.1016/j.biortech.2009.07.010

[92] Cao L, Jiang M, Zeng Z, Du A, Tan H, Liu Y (2008) Trichoderma atroviride F6 improves phytoextraction efficiency of mustard (Brassica juncea (L.) Coss. var. foliosa Bailey) in Cd, Ni contaminated soils. Chemosphere 71(9):1769–1773 18342911 10.1016/j.chemosphere.2008.01.066

[93] Taghavi Ghasemkheili F, Ekelund F, Johansen JL, Pirdashti H, Ghadirnezhad Shiade SR, Fathi A, Kjøller R (2022) Ameliorative effects of Trichoderma harzianum and rhizosphere soil microbes on cadmium biosorption of barley (Hordeum vulgare L.) in Cd-polluted soil. J Soil Sci Plant Nutr 22(1):527–539. 10.1007/s42729-021-00666-y

[94] Chanda D, Sharma GD, Jha DK, Hijri M (2017) Tolerance of microorganisms in soil contaminated with trace metals: an overview. Recent Adv Appl Microbiol 165–193. 10.1007/978-981-10-5275-0_8

[95] Hatvani L, Manczinger L, Kredics L, Szekeres A, Antal Z, Vágvölgyi C (2006) Production of *Trichoderma* strains with pesticide-polyresistance by mutagenesis and protoplast fusion. Antonie Van Leeuwenhoek 89(3):387–393 16779635 10.1007/s10482-005-9042-x

[96] Ezzi MI, Lynch JM (2002) Cyanide catabolizing enzymes in *Trichoderma* spp. Enzyme Microb Technol 31(7):1042–1047

[97] Elkhateeb WA, Elnahas MO, Daba GM, Zohri ANA (2021) Biotechnology and environmental applications of *Trichoderma* spp. Res J Pharmacogn Phytochem 13(3):149–157

[98] Van Gestel K, Mergaert J, Swings J, Coosemans J, Ryckeboer J (2003) Bioremediation of diesel oil-contaminated soil by composting with biowaste. Environ Pollut 125(3):361–368. 10.1016/S0269-7491(03)00109-X 12826414 10.1016/s0269-7491(03)00109-x

[99] Katayama A, Matsumura F (1993) Degradation of organochlorine pesticides, particularly endosulfan, by *Trichoderma harzianum*. Environ Toxicol Chem: Int J 12(6):1059–1065. 10.1002/etc.5620120607

[100] Sharma P, Kumar V, Ramesh, R, Saravanan, K, Deep, S, Sharma, M, …, Dinesh S (2011) Biocontrol genes from Trichoderma species: a review. Afr J Biotechnol 10(86):19898–19907. 10.5897/AJB11.2359

[101] Elshahawy IE, El-Mohamedy RS (2019) Biological control of Pythium damping-off and root-rot diseases of tomato using *Trichoderma* isolates employed alone or in combination. J Plant Pathol 101:597–608. 10.1007/s42161-019-00426-w

[102] Tripathi P, Singh PC, Mishra A, Chauhan PS, Dwivedi S, Bais RT, Tripathi RD (2013) *Trichoderma*: a potential bioremediator for environmental clean-up. Clean Technol Environ Policy 15(4):541–550. 10.1007/s10098-012-0521-5

[103] Manjunatha SV, Naik MK, Khan MFR, Goswami RS (2013) Evaluation of bio-control agents for management of dry root rot of chickpea caused by *Macrophomina phaseolina*. Crop Prot 45:147–150. 10.1016/j.cropro.2012.11.001

[104] Ferrigo D, Raiola A, Piccolo E, Scopel C, Causin RJJPP (2014) Trichoderma harzianum T22 induces in maize systemic resistance against Fusarium verticillioides. J Plant Pathol 96(1). 10.4454/jpp.v96i1.026

[105] Gao P, Qi K, Han Y, Ma L, Zhang B, Zhang Y, …, Qi J (2023) Effect of Trichoderma viride on rhizosphere microbial communities and biocontrol of soybean root rot. Front Microbiol 14:1204688. 10.3389/fmicb.2023.1204688 10.3389/fmicb.2023.1204688 PMC10272447 37333630

[106] Ghazanfar MU, Raza M, Raza W, Qamar MI (2018) *Trichoderma* as potential biocontrol agent, its exploitation in agriculture: a review. Plant Protect 2(3):109–13. 10.11648/j.pp.20180203.12

[107] Sharma P, Sharma M, Raja M, Shanmugam V (2014) Status of *Trichoderma* research in India: a review. Indian Phytopathol 67(1):1–19. 10.1007/s42360-019-00001-w

[108] Reddy PY, Jakhar SS, Dahiya OS (2019) Management of fruit rot of Chilli caused by *Colletotrichum capsici*. Int J Curr Microbiol Appl Sci 8(5):523–538. 10.20546/ijcmas.2019.805.068

[109] Prasad RD, Rangeshwaran R, Hegde SV, Anuroop CP (2002) Effect of soil and seed application of *Trichoderma harzianum* on pigeonpea wilt caused by *Fusarium udum* under field conditions. Crop Prot 21(4):293–297. 10.1016/S0261-2194(01)00107-4

[110] El-Hassan SA, Gowen SR, Pembroke B (2013) Use of Trichoderma hamatum for biocontrol of lentil vascular wilt disease: efficacy, mechanisms of interaction and future prospects. J Plant Protect Res 53(1). 10.2478/jppr-2013-0001

[111] Ejechi BO (1997) Biological control of wood decay in an open tropical environment with *Penicillium* sp. and *Trichoderma**viride*. Int Biodeterior Biodegr 39(4):295–299. 10.1016/S0964-8305(97)00001-6

[112] Kundu A, Chakraborty MR, Chatterjee NC (2008) Biocontrol of wood decay by *Trichoderma* spp.–retrospect and prospect. Asian J Exp Sci 22(3):373–384. 10.1007/s12150-008-0058-1

[113] Fojutowski A, Kropacz A, Koziróg A (2016) The growth of *Trichoderma viride* fungus on an agar and wood substrate. J Appl Microbiol 120(3):734–741. 10.1111/jam.13016

[114] Hidangmayum A, Dwivedi P (2018) Plant responses to *Trichoderma* spp. and their tolerance to abiotic stresses: a review. J Pharmacogn Phytochem 7(1):758–766. 10.5958/0975-4426.2018.00098.5

[115] Sawant IS, Mukhopadhyay AN (1991) Integration of metalaxyl with *Trichoderma harzianum* for the control of *Pythium* damping-off in sugarbeet. Indian Phytopathol 44(2):149–154. 10.1007/BF02951412

[116] Oros G, Naár Z, Cserháti T (2011) Growth response of *Trichoderma* species to organic solvents. Mol Inf 30(2–3):276–285. 10.1002/minf.201000083 10.1002/minf.201000097 27466781

